# Smartphone-Delivered Peer Physical Activity Counseling Program for Individuals With Spinal Cord Injury: Protocol for Development and Pilot Evaluation

**DOI:** 10.2196/10798

**Published:** 2019-03-22

**Authors:** Krista L Best, François Routhier, Shane N Sweet, Emilie Lacroix, Kelly P Arbour-Nicitopoulos, Jaimie F Borisoff

**Affiliations:** 1 Centre for Interdisciplinary Research in Rehabilitation and Social Integration Institut de Réadaptation en Déficience Physique de Québec Centre Intégré Universitaire de Santé et de Services Sociaux de la Capitale National Quebec, QC Canada; 2 Department of Rehabilitation Faculty of Medicine Université Laval Quebec, QC Canada; 3 Department of Kinesiology & Physical Education McGill University Montreal, QC Canada; 4 Centre de Recherche Interdisciplinaire en Réadaptation du Montréal Métropolitain Montreal, QC Canada; 5 Faculty of Kinesiology and Physical Education University of Toronto Toronto, ON Canada; 6 Rehabilitation Engineering Design Laboratory British Columbia Institute of Technology Burnaby, BC Canada; 7 International Collaboration on Repair Discoveries Vancouver Coastal Health Vancouver, BC Canada

**Keywords:** smartphone, mobile phone, behavior change, digital peer training, leisure-time physical activity, spinal cord injury, Medical Research Council framework

## Abstract

**Background:**

Leisure-time physical activity (LTPA) is a critical component of a healthy lifestyle for individuals with spinal cord injury (SCI). However, most individuals are not sufficiently active to accrue health benefits. The Active Living Lifestyles program for individuals with SCI who use manual wheelchairs (ALLWheel) targets important psychological factors that are associated with LTPA uptake and adherence while overcoming some barriers associated with participation restrictions.

**Objective:**

The goal of the paper is to describe the protocol for the development and evaluation of the ALLWheel program for individuals with SCI who use manual wheelchairs.

**Methods:**

The first three stages of the Medical Research Council framework for developing and evaluating complex interventions (ie, preclinical, modeling, exploratory) are described. The preclinical phase will consist of scoping and systematic reviews and review of theory. The intervention will be modeled by expert opinions and consensus through focus groups and Delphi surveys with individuals with SCI, clinicians, and community partners. Finally, the feasibility and potential influence of the ALLWheel program on LTPA and psychological outcomes will be evaluated.

**Results:**

This project is funded by the Craig H Neilsen Foundation, the Fonds de Recherche du Québec–Santé, and the Canadian Disability Participation Project and is currently underway.

**Conclusions:**

Using peer trainers and mobile phone technology may help to cultivate autonomy-supportive environments that also enhance self-efficacy. Following a framework for developing and evaluating a novel intervention that includes input from stakeholders at all stages will ensure the final product (ie, a replicable intervention) is desirable to knowledge users and ready for evaluation in a randomized controlled trial. If effective, the ALLWheel program has the potential to reach a large number of individuals with SCI to promote LTPA uptake and adherence.

**International Registered Report Identifier (IRRID):**

DERR1-10.2196/10798

## Introduction

### Leisure-Time Physical Activity Is Critical for Individuals With Spinal Cord Injury

Spinal cord injury (SCI) is associated with various sequelae, including respiratory disease, heart disease, diabetes, osteoporosis, overuse injuries, sexual disorders, pressure ulcers, chronic pain, fatigue, and depression [1,2]. Furthermore, the increased risk of sedentariness that often results from reduced mobility after SCI (eg, sitting in a wheelchair) can trigger a chain of negative physiological and psychological events that exacerbate secondary health conditions [3].

Participation in leisure-time physical activity (LTPA) can have a profound impact on health and quality of life after SCI. From a physiological perspective, findings from two systematic reviews confirm that participation in LTPA improves physical capacity, muscular strength, and respiratory function [4] and lowers risk factors associated with endocrine metabolic disease (eg, heart disease, osteoporosis, diabetes) [5]. Evidence from three systematic reviews suggests that participation in regular LTPA can positively influence psychosocial factors, including motivation, quality of life, and overall well-being [6-8].

Participation in LTPA is critical for individuals with SCI [8], especially those who use wheelchairs [9]. Even moderate amounts of LTPA may optimize functioning and slow the spiraling effects of deconditioning that are associated with SCI [10]. It is promising that many individuals with SCI have high LTPA intentions [11]. However, it is concerning that most are not active enough to accrue the health benefits. The results of two surveys with 73 and 965 individuals with SCI highlight this problem, reporting that 45% to 50% of respondents did not participate in any LTPA at all [11,12]. Therefore, the medical community is being encouraged to consider LTPA as a critical outcome that needs to be monitored for individuals with SCI [13].

### Approaches to Community-Based Leisure-Time Physical Activity for Individuals With Spinal Cord Injury

Compared to the general population, individuals with SCI find it more difficult to start and adhere to LTPA regimes due to physical, environmental, and psychological barriers [13-16]. Transportation and physical health were the most commonly reported barriers [17]. Several approaches have been shown to be feasible and successful for overcoming the barriers and improving LTPA among individuals with SCI in the community, including home visits [18], telephone-delivered programs [19-22], online support [23], and gamification and virtual reality [24,25]. However, many existing approaches lack a strong grounding in behavior change theory [23] and thus may miss important psychological factors that are known to influence LTPA behavior over the long term. Telephone-delivered interventions represent one approach with a strong theoretical foundation that has been shown to sustain LTPA intentions over time [19,20]. Moreover, telephones have been reported as the preferred method of intervention delivery among individuals with SCI [26].

While telephone counseling presents a promising strategy for promoting LTPA among adults with SCI, direct and continuous contact has been reported to be important for enhancing effectiveness and adherence [27]. Therefore, LTPA programs for individuals with SCI should maintain the advantages of telephone delivery to overcome some of the barriers to LTPA but also integrate face-to-face contact.

### Psychological Factors Influencing Leisure-Time Physical Activity Behavior

There are important psychological factors that influence LTPA uptake, adherence, and retention that need to be considered including autonomy support, motivation, and self-efficacy [28]. Self-determination theory provides a framework for understanding the motivations that may influence change in LTPA behavior [29] and has been effectively applied in the development of LTPA interventions [30]. Self-determination theory posits that through the satisfaction of autonomy, competence, and relatedness [31], autonomous motivation (ie, engaging in an activity for the value, importance, or enjoyment of the behavior) is increased and subsequently drives behavior change and maintenance [32]. Perceived competence, a similar construct to self-efficacy (ie, an individual’s belief in his or her ability to accomplish a specific task [33]), has been shown to be a key determinant in eliciting LTPA behavior change [32]. In fact, self-efficacy is one of the most salient factors predicting uptake and maintenance of LTPA [34,35].

Peers are particularly useful role models after SCI, as they can help to establish a meaningful social network through shared life experiences, relatedness, and management of similar conditions [36-38]. Intervention delivery by peers can provide a source of personal contact (eg, face-to-face contact), which has been shown to increase LTPA and satisfaction with participation among individuals with SCI [18,39,40]. Although peers represent an influential approach to enhance self-efficacy and motivation for LTPA, existing programs have not fully incorporated the use of the power of SCI peers [39,40].

### Mobile Technology and Social Media

Mobile technology (ie, smartphones and tablets) are becoming ubiquitous and may afford greater accessibility and convenience for the SCI population to participate in LTPA interventions [41,42]. Advancements and access to mobile technology may also extend the reach and effectiveness of telephone-delivered interventions. For instance, social networking available through smartphones and tablets may offer increased methods for achieving personal contact (eg, contact with peer groups) and may improve solutions to the timely delivery of LTPA interventions for individuals with SCI [43]. Importantly, the use of mobile technology and social media to deliver LTPA interventions can allow for various methods of contact depending on participant preferences (eg, voice and video calls, text messaging).

Smartphones represent an affordable, portable, and novel approach using modern technology that may provide a useful medium for integrating important psychological variables (supportive environment, motivation, self-efficacy) while providing remote access to an LTPA intervention that is designed specifically for individuals with SCI. Integrating peers to deliver the LTPA program adds an important social element that may further enhance motivation and self-efficacy. However, given that 33% to 50% of individuals with SCI may not be able to use mobile technology [44], an LTPA program delivered by peers through social networking may offer alternate ways to access the program, including from desktop and laptop computers, which could accommodate various needs.

The aim of this paper is to describe the protocol for the development and evaluation of a theory-informed Active Living Lifestyles program for individuals with SCI who use a manual wheelchair (ALLWheel). In its early conceptualization, the name of the program was the Smartphone-Delivered Peer Physical Activity Counseling (SPPAC) program. Given the evolution of the program, the name ALLWheel will be used in all future evaluation and dissemination.

## Methods

### Guiding Framework

**Figure 1:**
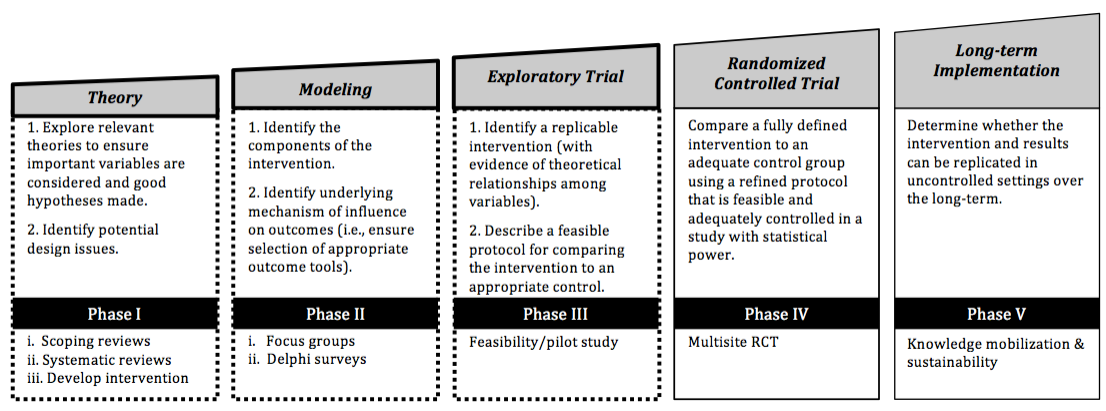
Illustration of the processes for the development and evaluation of the ALLWheel program according to the Medical Research Council framework. RCT: randomized controlled trial.

The Medical Research Council methodological framework was applied to design the protocol for the development and evaluation of the ALLWheel program [45]. The Medical Research Council framework describes five distinct phases: preclinical or theoretical (phase I), modeling (phase II), exploratory (phase III), randomized controlled trial (RCT) (phase IV), and long-term implementation (phase V) [45]. Figure 1 illustrates the Medical Research Council framework, highlighting phases I to III.

### Phase I: Preclinical and Theory

#### Objective Ia

The objectives of this study are to summarize the impact of existing LTPA programs in Canada, identify existing gaps in programming for individuals with SCI, and make recommendations to address some of the gaps.

##### Design

Scoping reviews provide a form of knowledge synthesis that addresses an exploratory research question to map key concepts, summarize evidence, and identify gaps in research [46].

##### Procedure

Three experts in SCI and LTPA will follow a 6-step approach: identification of the research question, identification of relevant articles, article selection, evidence extraction, synthesizing and summarizing the data, and consultation with stakeholders [47]. The review will consist of (1) a systematic search of the scientific literature (ie, electronic databases including PubMed/MEDLINE, PsycINFO, CINAHL) using key words for spinal cord injury, physical activity, mobility, and community and (2) a Google search based on the authors’ knowledge of existing programs and the abovementioned keywords. All scientific and grey literature pertinent to the objective will be considered. Findings from this scoping review will be used to design a subsequent systematic review (objective Ib) and to develop a focus group schedule (objective IIa) [48].

#### Objective Ib

The primary objective of this study is to determine the effectiveness of existing programs on LTPA among individuals with SCI who use manual wheelchairs. Secondary objectives include summarizing details related to program content, delivery methods, integration, and facilitators and barriers and discussing the potential of a mobile phone and peer-led LTPA program for overcoming some of the barriers reported among individuals with SCI.

##### Design

A systematic review will be done according to the Preferred Reporting Items for Systematic reviews and Meta-Analyses statement guidelines (prisma-statement.org) [49,50].

##### Procedure

Original searches will be conducted by two independent researchers using online databases (PubMed/MEDLINE, CINAHL, PsycINFO, Embase, SPORTDiscus). Reference lists of selected studies and relevant review articles will be hand searched. The search strategy and study selection criteria will be developed according to the Participant, Intervention, Comparison, and Outcomes guidelines as described in the Cochrane Handbook for Systematic Reviews [51]. Accordingly, keywords will include terms relevant to Participant (spinal cord injury, manual wheelchairs), Intervention (LTPA, physical activity, self-determination theory, social cognitive theory, behavior change), Comparison (randomized controlled trial, quasi-experimental), and Outcomes (physical activity, participation, motivation, self-efficacy). Studies that fit the Cochrane guidelines and are written in English will be included in the review.

Two reviewers will independently rate the titles, abstracts, and full texts and select articles for inclusion. If consensus is not reached regarding inclusion criteria, a third reviewer will be consulted. The same two reviewers will assess methodological quality of randomized controlled trials using the Physical Therapy Evidence Database [52] and of pre-post studies using the Quality Assessment Tool for Before-After (Pre-Post) Studies With No Control Group [53]. Relevant data (mean differences in LTPA from pre- to immediately post-intervention) will be extracted to meet the primary objective. If feasible, a meta-analysis will be conducted. To meet the secondary objective, details about the program content, delivery methods, facilitators and barriers, and other pertinent information will be extracted and organized according to the two theoretical frameworks (ie, self-determination theory [29,33] and social cognitive theory [33]) that guide this research. Findings from this review will be used to design the prototype for a new LTPA intervention and develop discussion points for the focus group schedule.

### Phase II: Modeling

#### Objective IIa

The first objective in the modeling phase is to gain expert input about the initial prototype of a novel LTPA program.

##### Design

A qualitative study design will be used.

##### Participant Recruitment

Purposive sampling will be used to recruit 10 to 15 experts (ie, individuals with SCI, health care professionals, and community partners that specialize in SCI) to participate in a focus group. Involving key stakeholders has been shown to improve intervention development and outcome selection [54]. Health care professionals will be kinesiologists, occupational therapists, and physiotherapists who have at least 5 years’ experience working with the SCI population or at least 5 years of experience with LTPA interventions for persons with SCI. Individuals with SCI will be eligible to participate if they are aged 18 years and older, live in the community, have a traumatic or nontraumatic SCI (tetraplegia or paraplegia), use a manual wheelchair as their primary means of mobility, and are one or more years post-SCI [55]. Participants will be identified through existing community partners (eg, Adaptavie, Viomax, and Spinal Cord Injury British Columbia) and clinical partners (eg, outpatient rehabilitation programs at each site) that are the knowledge users of the ALLWheel program. Institutional ethics guidelines will be followed and informed consent will be obtained.

##### Procedure

Based on the findings from phase I, a concept version of the ALLWheel program including intervention content and smartphone apps (eg, voice calls, text messaging, videoconference, social media) will be provided to participants before the focus group. Participants will also receive an open-ended questionnaire to complete prior to the focus group where they will be asked to provide descriptive information about appropriateness of the ALLWheel intervention, suggestions for changes to content or delivery method, missing content, and potential concerns. Responses from the questionnaires will be used to guide the discussion during the focus groups. The focus group guide will be developed according to the Interview Protocol Refinement framework such that questions will align with the study objectives, questions will be organized to create an inquiry-based conversation, and the protocol will be reviewed and piloted among the research team [56]. A moderator and a research assistant will open the discussion with a brief description of results from the questionnaire and facilitate a brainstorming activity to determine potential modifications to the ALLWheel intervention protocol. Two focus groups will be conducted (each with 6 to 8 knowledge users who will be individuals with SCI, members of community groups, and clinicians) over 90 minutes and will be audiorecorded.

#### Data Analysis

Summary statistics will be used to describe the sample. Audiorecordings from the focus groups will be transcribed verbatim and analyzed using NVivo qualitative data analysis version 10 software (QSR International Pty Ltd). Content analysis will be done to identify recommendations for modifications to intervention delivery method or content, additional content to be included, and appropriateness of outcome measurement and determine if other general changes are necessary. Two to three individuals will perform a directed content analysis by repeatedly reviewing and organizing the data and extracting meaningful units into major themes and subthemes. Themes and subthemes will be discussed and agreed upon by the research team, and findings will be presented to the subjects in the form of Delphi surveys to obtain consensus.

#### Objective IIb

The second objective of the modeling phase is to achieve consensus from experts on a novel LTPA program.

##### Design

The Delphi method is a widely accepted and useful research approach for intervention development in the absence of sufficient evidence from experimental studies [57]. A group of experts provide insight on the topic through sequential questioning (ie, in multiple rounds) until consensus is obtained among the group [58,59]. In this way, the Delphi method will provide useful insight about an LTPA intervention that uses peers and smartphone technology for individuals with SCI who use manual wheelchairs.

##### Participant Recruitment

The same individuals who participated in the focus groups will form an expert panel and complete the Delphi surveys.

##### Procedure

Using an iterative process, participants will complete written questionnaires in multiple rounds to achieve consensus on the ALLWheel intervention structure and content [60]. In the first Delphi round, the ALLWheel intervention will be presented and experts will be asked to provide anonymous feedback. Details of the ALLWheel intervention will be described (eg, components to be included, useful motivational strategies, preferred program delivery methods, critical considerations) using definitive statements, and participants will rate their level of agreement or importance using Likert scales (eg, strongly agree to strongly disagree; not at all important to very important). Participants will then be asked for additional suggestions for improvement using open-ended questions. Subsequent Delphi rounds will be administered until 70% consensus is achieved [57]. The final step will consist of an expert meeting to integrate findings from the Delphi survey (eg, components to include/exclude, delivery methods preferred, critical motivation strategies) to generate a concept version of the ALLWheel program [57].

### Phase III: Exploratory Trial

#### Objective IIIa

The first exploratory objective is to evaluate the feasibility of the ALLWheel study protocol for indicators of process, resources, management and treatment effect.

#### Objective IIIb

The second exploratory objective is to evaluate the influence of ALLWheel on objective LTPA (actigraphy).

#### Objective IIIc

The third exploratory objective is to evaluate the influence of ALLWheel on subjective LTPA, barriers to LTPA, motivation, psychological needs satisfaction, and satisfaction with participation.

#### Objective IIId

The fourth exploratory objective is to explore potential mediating and moderating relationships between LTPA and sociodemographic factors, epidemiological variables, and all secondary outcomes.

##### Design

A three-site, pre-post feasibility study will be done.

##### Participant Recruitment

A convenience sample of 12 community-dwelling individuals living with SCI will be recruited through community partners (eg, Adaptavie, Viomax, and Spinal Cord Injury British Columbia) and clinical partners (eg, outpatient rehabilitation programs at each site) that are the knowledge users of the ALLWheel program and who will be involved in the development and refinement of the program. According to Hertzog [61], 10 to 15 participants may be adequate to detect feasibility in a pilot study. Since the primary purpose of this study is to assess feasibility of the ALLWheel protocol for a future clinical trial, from a pragmatic perspective of future recruitment in a relatively small population, a smaller sample size is justifiable. Participants will be between 18 and 65 years old, live in the community, have had an SCI for 1 or more years [55], use a manual wheelchair as their primary means of mobility, be able to self-propel a manual wheelchair for at least 100 meters; not currently be meeting the physical activity recommendations [62], and be cognitively able to engage in the ALLWheel intervention (Mini-Mental State Exam score ≥25) [63]. Individuals will be excluded if they anticipate a health condition or procedure that contraindicates training, have a degenerative condition that is expected to progress quickly, or are concurrently or planning to take part in another LTPA intervention over the period of the study. Participants will be screened using the Physical Activity Readiness Questionnaire and e-PARmed-X+ [64]. Institutional ethics guidelines will be followed at each of the three sites, and participants will provide informed consent.

##### Procedure

Preferred duration and delivery methods for the ALLWheel intervention will be explored in phase II. However, for the purposes of study planning and budgeting, the intervention length (ie, 6 months) and number of contacts with participants (ie, 14) will be based on the findings of an effective telephone-counseling intervention for improving LTPA for individuals with SCI [19]. Program sessions will be customized to individual LTPA goals, and spouses/partners may be integrated into the ALLWheel program if desired by participants. For the proposed study, a physically active peer coach who has had an SCI for at least 5 years will deliver the ALLWheel intervention. The peer coach will receive comprehensive training through a 2- to 3-day workshop administered by study investigators.

#### Outcome Measures

All assessments will be administered by trained testers at each site who will be trained in a 3-hour workshop facilitated by study investigators (KB, EL).

Descriptive characteristics and sociodemographic information that are known to influence LTPA among individuals with SCI will be collected at baseline (T1) including age, sex, marital status, income, level of SCI, medications, psychological well-being, and social support [9,65-67]. Depression and anxiety will be assessed using the 14-item Hospital Anxiety and Depression Score [68,69], and social support will be assessed using the 6-item Interpersonal Support Evaluation List [70,71].

##### Feasibility Indicators

Feasibility indicators related to process, resources, management, and treatment will be collected throughout study [72]. A description of feasibility indicators, how they will be measured, and the parameters for success are described in Multimedia Appendix 1. Testers at each site will administer all outcome measures at baseline (T1), postintervention (T2), and 3 months postintervention (T3). The selected outcomes are reflective of important theoretical variables known to influence LTPA uptake, adherence, and retention. Additional outcomes may be identified during phases I and II.

##### Actigraphy

The primary outcome, LTPA, will be measured objectively using actigraphy, a noninvasive method of monitoring human activity using a small and lightweight accelerometry-based activity monitor (Actigraph GT3X+, ActiGraph LLC) that can be worn on the body of the wheelchair user and on the wheelchair without impeding movement [73]. The monitor contains an accelerometer that is sensitive to motion in all directions, and data are stored in the monitor as activity counts [74]. Time between sampling units (epochs) will be set at 15 seconds, allowing the greatest sensitivity for low-intensity activity [74]. Concurrent validity and reliability have been established [75,76]. Further validation for the use of actigraphy to distinguish between low and moderate intensities of LTPA among individual manual wheelchair users, including manual wheelchair users with SCI, is available elsewhere [77].

Upon completion of all secondary outcomes (subjective self-reports) at each time point, the tester will provide participants with 2 actigraphs (one will be positioned on the rear wheel of the manual wheelchair in a waterproof enclosure; the other will be worn on the nondominant arm). Participants will be asked to wear the actigraph at all times over a 7-day period except during sleep, bathing, or swimming. Participants will record the time the actigraph was put on and taken off using a log. The tester will obtain the actigraph and log from the participant at the end of the 7-day period. Only data from the days in which the actigraphs are worn for at least 13 hours per day will be included in the analysis [78]. Data will be converted to mean activity counts per hour (ie, bouts per hour).

Secondary outcomes reflect the proposed theoretical impacts of the ALLWheel intervention (ie, the relationship between LTPA behavior) and psychological determinants of behavior change (eg, motivation, autonomy support, and satisfaction of psychological need for LTPA). The secondary outcomes will help to discern a clinically important impact of the ALLWheel intervention.

##### Leisure-Time Physical Activity Questionnaire

Self-reported LTPA behavior will be measured using the 7-day Leisure-Time Physical Activity Questionnaire for adults with SCI [79]. Since actigraphy may not capture the intensity of some activities (eg, weightlifting) and they cannot be worn while swimming, participants will also be asked to recall the frequency (number of bouts) and duration (minutes per bout) of light, moderate, and heavy intensity LTPA over the past 7 days. Acceptable test-retest reliability and construct validity have been documented among adults with SCI [80,81].

##### Treatment Self-Regulation Questionnaire

Motivation to participate in LTPA will be evaluated using the 15-item Treatment Self-Regulation Questionnaire [82], which is designed to measure the degree of autonomous self-regulation to participate in healthy behaviors. Reasons for engaging in or changing health behaviors are scored using a 7-point Likert scale ranging from 1 (not true at all) to 7 (very true). Three subscales assess 6 forms of motivation, including autonomous regulation (identified, integrated, and intrinsic motivations), controlled regulation (external and introjected motivations), and amotivation. The questionnaire has been validated for assessing motivation for engaging in exercise [83]. Since the purpose of this study is to assess participation in physical activity that one engages in during their free time, wording for *exercise* will be changed to LTPA.

##### Leisure-Time Barrier Self-Efficacy Scale

Self-efficacy to overcome salient barriers to LTPA participation (eg, transportation problems, bad weather, pain, fatigue) will be assessed using a 6-item Leisure-Time Barrier Self-Efficacy Scale. The scale has been used in previous research with SCI [84-86] with evidence of high reliability and validity [84] and acceptable internal consistency [81].

##### Psychological Need Satisfaction in Exercise Scale

Satisfaction of the psychological needs for LTPA will be assessed using the Psychological Need Satisfaction in Exercise Scale [87]. Participants are asked to rate 18 items that reflect how a person may feel during physical activity using a 6-point Likert scale. A mean score will be calculated for autonomy, competence, and relatedness.

##### Wheelchair Outcome Measure

The Wheelchair Outcome Measure is a semistructured interview that allows participants to select important wheelchair-oriented participation goals. Participants are asked to identify 2 to 5 goals and evaluate their current satisfaction with participation in each goal (on a scale from 0 to 10). Participation goals are incorporated into the intervention. The instrument demonstrates good reliability and validity in use among individuals with SCI and older adults [88,89].

#### Data Analysis

Analyses will consider study feasibility indicators and primary and secondary outcomes. Means and standard deviations (continuous variables) and frequencies and proportions (categorical variables) will be used to summarize all data. Feasibility outcomes (objective IIIa) will be treated as binary, with *success* indicating the protocol is sufficiently robust to move forward with an RCT with only small or no adaptation required and *revise* indicating a need for changes before proceeding (see Multimedia Appendix 1). Controlling for confounding and within-subject changes from baseline to postintervention and from baseline to follow-up in LTPA behavior will be determined using paired sample *t* tests (or nonparametric equivalent) (objective IIIb). Paired sample *t* tests will be used to evaluate within-subject change scores from baseline to postintervention and from baseline to follow-up for self-reported LTPA, motivation, LTPA barrier self-efficacy, autonomy support, satisfaction of the psychological needs for LTPA, satisfaction with participation in meaningful activities, and controlling for confounding (objective IIIc). Exploratory analyses (objective IIId) will be conducted to investigate the strength and direction of the relationships between sociodemographic and epidemiological factors and primary and secondary outcomes, looking for moderate to strong relationships [90].

## Results

This project is funded by the Craig H Neilsen Foundation, the Fonds de Recherche du Québec–Santé, and the Canadian Disability Participation Project. Approval has been obtained from the university research ethics boards at all sites for all phases of the study. Phase I scoping and systematic reviews have been completed, and manuscript preparation is underway. Phase II focus groups and Delphi surveys are near completion, and manuscript preparation is underway. Phase III pilot and feasibility evaluation is currently underway. All study staff have been hired and trained at all sites, and recruitment and data collection are ongoing. Four peer trainers have been recruited and trained, and recruitment for phase III was completed in September 2018.

## Discussion

### Principal Findings

The ALLWheel intervention presents an innovative approach to targeting change in LTPA for individuals with SCI. Guided by the tenets of two behavior change theories (ie, self-determination theory and social cognitive theory), conception of ALLWheel will integrate important psychological precursors to LTPA including autonomy, relatedness, competence/self-efficacy, and motivation [29-33]. Furthermore, development of the ALLWheel intervention and study protocol will follow the Medical Research Council framework for developing and evaluating complex interventions [45], which will ensure that ALLWheel is evidence-based. Development of the ALLWheel program will also involve knowledge users (eg, individuals with SCI, community organizations, clinicians) throughout all aspects of development, evaluation, and implementation, ensuring an integrated approach to knowledge translation. Finally, a feasibility evaluation will allow for refinement of the intervention and iterations of the protocol to maximize its impact.

Although the LTPA needs of individuals with SCI are not fully understood, there is reason to believe that including peers in intervention delivery may have benefits [39]. Furthermore, a program delivered using a smartphone has the potential to overcome many existing barriers to LTPA for individuals with SCI and allows for integration of an important face-to-face component (ie, through video-conferencing). The application of digital peer training (ie, digital person-to-person training facilitated by a peer using smartphone technology [91]) could maintain the benefits of telephone-delivered interventions (eg, increased geographic reach [19-21]), incorporate human support (ie, an important predictor of effect and adherence of behavior change interventions [91]), ensure individually tailored programs, and facilitate the implementation of important psychological factors [31-33]. Evaluating outcomes of autonomy, motivation, and self-efficacy will allow for exploration of theorized relationship between psychological factors and LTPA, which will provide crucial information for refinement of the intervention before conducting a larger RCT.

Including expert stakeholders (ie, individuals with SCI, clinicians, and community partners) in the development of a theory-based ALLWheel intervention is an integral component of this research program [54]. Obtaining consensus from our stakeholders and knowledge users will ensure that we develop a comprehensive LTPA intervention that is desirable to the people for whom it is intended. Evaluating the feasibility of the intervention in pre-post study design will allow for feedback from the stakeholders and modifications before implementing a larger more expensive effectiveness trial.

ALLWheel has potential for large geographic reach to individuals of various ages, and determining the feasibility of administering the program in English and French may lead to translation in other commonly used languages in Canada. Future studies can estimate cost effectiveness, measure long-term retention, and assess impact on the known health benefits.

### Limitations

Larger multisite clinical trials are required to establish evidence that informs effective behavior change strategies for individuals with SCI. However, a 3-year development and feasibility study is a critical and prudent process to follow before designing a large and expensive multisite RCT. Developing a pilot and testing the intervention according to the Medical Research Council framework will help to ensure that the intervention is evidence-based and the protocol and intervention are feasible to administer. While the generalizability of ALLWheel is limited to individuals with SCI at this point, it is possible that digital peer training may provide a useful strategy for delivering LTPA programs to a broader population of wheelchair users and even the general population.

### Conclusion

Using peer coaches and smartphone technology may help to cultivate autonomy supportive environments that also enhance self-efficacy. Following a framework for developing and evaluating a novel intervention that includes input from stakeholders at all stages will ensure the final product (ie, a replicable intervention) is desirable to knowledge users and ready for evaluation in an RCT. If effective, the ALLWheel program has the potential to reach a large number of individuals with SCI to promote LTPA uptake and adherence.

## Appendix

Multimedia Appendix 1 Description of feasibility indicators and parameters for the success of the Smartphone-Delivered Peer Physical Activity Counseling intervention and study protocol.

Multimedia Appendix 2 Peer-reviewer report from the Craig H Neilsen Foundation.

## Abbreviations

ALLWheel: Active Living Lifestyles for Individuals Who Use Wheelchairs
LTPA: leisure-time physical activity
RCT: randomized controlled trial
SCI: spinal cord injury
SPPAC: Smartphone-Delivered Peer Physical Activity Counseling
