# Supplementary material for: Smartphone-Delivered Peer Physical Activity Counseling Program for Individuals With Spinal Cord Injury: Protocol for Development and Pilot Evaluation
Source: JMIR Res Protoc. 2019 Mar 22;8(3):e10798. doi: 10.2196/10798 (PMC6450480; doi:10.2196/10798)
Supplement: Multimedia Appendix 1 [file resprot_v8i3e10798_app1.pdf]

Multimedia Appendix 1. Description of feasibility indicators, and parameters for success of the SPPAC intervention and study protocol.

Feasibility indicator	Outcome measure	Parameter for success
Process		
Recruitment rate	# of participants recruited/ time	2 participants/month
Consent rate	% of participants consenting	> 20% acceptance
Retention rate	% of participants with complete data at T2 and T3	Complete T2 & T3 with ≥ 80% of participants
Perceived benefit	Post-intervention interview	Qualitative analysis
Resources		
Participant adherence	Complete 14 SPPAC sessions	> 85% of participants
Peer-trainer adherence	Recruit /retain peer-trainers	Facilitate 14 x12 sessions
Data collection burden		
T1	Data collection time T1	> 85% of participants complete in ≤ 2 h
T2	Data collection time T2	> 85% of participants complete in ≤ 1.5 h
T3	Data collection time T3	> 85% of participants complete in ≤ 1.5 h
Translations	Translate and administer study materials in English and French	No issues
Management		
Processing time	Time between initial contact to enrolment	Mean time is < 30 days
Combining data	Combine data in English and French	No issues
Protocol administration	Study protocol checklist	Modifications can be made with minimal changes
Intervention fidelity	Peer-trainer SPPAC checklist	Peer-trainer completes >85% of checklist
	Health Care Climate Questionnaire*	Participants will rate their perceived autonomy support as 5 points on average at T2 and T3
Safety		
Intervention	# of adverse events	No adverse events
Data collection	# of adverse events	No adverse events

* The Health Care Climate Questionnaire, a 6-item scale, will be used to assess perceived autonomy support [73]. Participants will be asked to respond to questions about their perceived PA autonomy on a 7-point Likert scale at mid-intervention (i.e., between session 6 and 7), and at T2 and T3. High Chronbach's alpha levels have been demonstrated in previous studies [73-74].

73. Williams GC, Grow VM, Freedman ZR, Ryan RM, Deci EL. Motivational predictors of weight loss and weight-loss maintenance. J Pers Soc Psychol 1996; 70(1):115–126.
74. Williams GC, McGregor HA, King D, Nelson CC, Glasgow RE. Variation in perceived competence, glycemic control, and patient satisfaction: Relationship to autonomy support from physicians. Patient Educ. Couns 2005; 57(1):39–45. PMID: 8558405
