# Supplementary material for: Smartphone-Delivered Peer Physical Activity Counseling Program for Individuals With Spinal Cord Injury: Protocol for Development and Pilot Evaluation
Source: JMIR Res Protoc. 2019 Mar 22;8(3):e10798. doi: 10.2196/10798 (PMC6450480; doi:10.2196/10798)
Supplement: Multimedia Appendix 2 [file resprot_v8i3e10798_app2.pdf]

NEILSEN FOUNDATION RESEARCH GRANT AGREEMENT

Grant Application ID Number: 366605

Grant Recipient Institution (the “Grantee”): Centre Intégré Universitaire de Santé et de Services Sociaux de la Capitale-Nationale

Principal Investigator: Krista Best, Ph.D.

Grant Title: Evaluation of a Smartphone-based Peer Counselling program for individuals with SCI

Final Grant Amount Approved	(Year 1):	$ 75,000	(“First Year Payment”)
	(Year 2):	$ 60,000	(“Second Year Payment”)
		$ 15,000	(“Final Payment”)
		$150,000	(“Total Grant Amount”)

Grant Term: April 30, 2016 - April 30, 2018

Report Deadlines:
Progress Report: April 30, 2017
Final Report: May 30, 2018

The Craig H. Neilsen Foundation (the “Foundation”) agrees to make the following grant, and the Grantee agrees to accept such grant, in accordance with the terms in this Neilsen Foundation Research Grant Agreement (this “Grant Agreement”) effective as of the date of the last signature hereon.

1. Use of Grant Funds The Grantee agrees that the funds granted by the Foundation will be used only for the purpose described in the grant application identified above (the “Application”), and the sections of the Application entitled “Aims,” “Abstract” and “Budget” (the “Incorporated Terms”) are hereby incorporated by reference into this Grant Agreement. Except as otherwise provided in the Incorporated Terms, neither the Grantee nor the Foundation has designated these grant funds or any portion thereof for any organization/institution or individual other than the Grantee. Any deviation from the Incorporated Terms with respect to the use of these grant funds must be approved in advance by the Foundation in writing.

2. Payment of Grant Funds The Total Grant Amount will be paid in three installments as follows:

   a. The First Year Payment will be made following receipt of this signed Grant Agreement, a Certification of Tax-Exempt Status form, and the required deliverables in the Award Management System of proposalCENTRAL. The Grantee will be notified separately of the deadlines for returning these materials.
   b. The Second Year Payment will be made following timely submission of the Progress and Interim Expenditure Report by the Grantee and review and approval of the reports by the Foundation.
   c. The Final Payment will be made following timely submission of the Final Research and Final Expenditure Report by the Grantee and review and approval of the final reports by the Foundation.
   d. Notwithstanding any other provision of this Grant Agreement, if the Grantee does not provide the required deliverables and/or the Progress and Interim Expenditure Report by the deadlines mandated by the Foundation, this Grant Agreement may be cancelled by the

Foundation, in the Foundation's sole discretion, at which time the Foundation's obligations under this Grant Agreement shall terminate.

3. Reversion of Grant If any portion of the grant funds is not used or committed for the purposes or in the timeframe authorized by the Foundation, the Grantee shall either: (a) request the Foundation's written authorization for a modification to the requirements of this Grant Agreement or an extension of time for the disbursement of funds, the approval of which will be in the Foundation's discretion, or (b) promptly return the amount not used or committed to the Foundation. Notwithstanding the foregoing, if the Grantee requests the Foundation's written authorization for a modification to the requirements of this Grant Agreement or an extension of time for the use of such funds, as applicable, and the Foundation approves such modification, the Grantee may retain such grant funds to be used in accordance with this Grant Agreement, as so modified. The Grantee acknowledges that the Foundation has sole discretion to accept or reject the Grantee's request for a modification to this Grant Agreement in accordance with this Paragraph 3.

4. Disclosure of Other Funding Sources The Grantee represents that it has not received funding for the purpose described in the Application (or a substantially similar purpose) from any other third party. If, during the period of time between the submission of the Application and the receipt of the Final Grant Amount Approved, the Grantee receives any funding for the purpose described in the Application (or a substantially similar purpose) from any individual or entity other than the Foundation, the Grantee shall disclose the source and amount of such funding within fourteen days of receipt of such funding. The Foundation reserves the right to reduce the Final Grant Amount Approved by the amount the Grantee receives from any other source.

5. Certification and Maintenance of Exempt Organization Status The Grantee represents that it has received and continues to hold its qualifications as one of the following: (a) a tax exempt organization under Internal Revenue Code Section 501(c)(3) and a public charity as defined by Internal Revenue Code Section 509(a); or (b) a political subsidiary of the United States or any state as defined in Internal Revenue Code 170(c)(1); or (c) the Canadian equivalent thereof. The Grantee hereby agrees it will immediately give written notice to the Foundation of any change in or challenge to its tax exempt status or becomes a private foundation under Internal Revenue Code Section 509(a).

6. Reporting The Grantee shall submit a written Progress and Interim Expenditure Report and a Final Research and Final Expenditure Report to the Foundation no later than the dates set forth on page one of this Grant Agreement. Such reports shall be completed on the forms provided in the Award Management System on proposalCENTRAL, and shall be submitted via proposalCENTRAL by the Grantee. Failure to provide either (or both) of these reports will result in the Grantee's inability to secure future grants for the organization/institution and/or the individual submitting the grant.

   In addition, any significant change to the status (such as investigation or suspension) of the required institutional IACUC approval (for animal protocols) or institutional IRB approval (for human subject research), or if the project is put on "clinical hold" by the FDA during the course of the award, must be reported to the Foundation within five business days. Funds may not be used for animal or human subject research unless a current institutional IACUC or IRB approval is in effect and on record with the Foundation.

7. Financial Review and Records The Foundation, may at its expense and on reasonable notice to the Grantee, inspect or audit, or have inspected or have audited by a third party selected by the Foundation, the records of the Grantee insofar as they relate to the activities funded by this grant. The Grantee hereby agrees it will maintain its books and records in such a manner that the receipts and expenditures of the grant funds will be shown separately on such books and records. In addition, the Grantee hereby agrees it will maintain records of receipts and expenditures of grant funds as well as copies of the reports submitted to the Foundation and

supporting documentation available to the Foundation for inspection at reasonable times from the time of acceptance of this grant until one year after the Final Research and Final Expenditure Report has been submitted to the Foundation.

8. Evaluation The Foundation may, at its expense and on reasonable notice to the Grantee, conduct an evaluation of operations under this Grant Agreement, which may include visits by representatives of the Foundation to observe the Grantee’s program procedures and operations and to discuss the program with the Grantee’s personnel.

9. Acknowledgement of Support and Grant Publicity All means of communications (i.e. scientific papers, articles, posters, press releases, etc.) dealing with the grant shall acknowledge the Foundation’s support. The Foundation may publish, whether on its website or otherwise, the Grantee’s name, amount of the grant, and a descriptive abstract of the purpose and use of the grant without the Grantee’s prior approval. The Foundation may not use the Grantee’s name for any commercial advertising or other commercial purpose. The Foundation’s prior written approval shall be obtained before the Grantee issues any news release, public announcement, or other publicity concerning the grant or the Foundation (“Proposed Statement”); provided, however, that if the Foundation does not approve or reject a Proposed Statement within five business days of the Grantee’s delivery to the Foundation of such Proposed Statement, such Proposed Statement shall be deemed approved by the Foundation. The Grantee need not obtain the Foundation’s prior approval prior to publishing a scientific or medical journal submission or publication (a “Scientific Publication”); provided, however, that the Grantee shall deliver to the Foundation a copy of any proposed Scientific Publication at least five business days prior to publishing said Scientific Publication.

10. Substitute Principal Investigator The Grantee acknowledges that the execution of the Grant Agreement is conditioned on the identity and efforts of the Principal Investigator. If, at any time, Principal Investigator is no longer employed by (or if the Principal Investigator is a volunteer or independent contractor, is no longer affiliated with) the Grantee, the Grantee must immediately notify the Foundation of such termination. The Foundation’s prior written approval shall be obtained before a substitute Principal Investigator is appointed. A request for approval of substitution of the Principal Investigator named in this Grant Agreement must be accompanied by a resume or CV and biography of the Principal Investigator, and if any modification to the Grant use or expenditures will result from the substitution of Principal Investigator, all of the following information in writing: a strong scientific justification related to the scientific project, including any proposed changes in scope, any other sources of support not previously disclosed, and any budget changes resulting from the proposed change. If the arrangements proposed by the Grantee, including the qualifications of any proposed replacement, are not acceptable to the Foundation, the grant may be suspended or terminated in the Foundation’s sole discretion. If the Grantee desires or the Foundation requires termination of the project due to the failure to agree upon a suitable substitute Principal Investigator or other alternate arrangement, the Grantee shall return any promptly return to the Foundation any grant funds not used or committed (see Paragraph 3).

11. Grant Term Modification In the event that the anticipated start date of the Grant Term is delayed by no fault of the Grantee or the Foundation during the Foundation’s due diligence process and prior to the submission of all required deliverables in the Award Management System of proposalCENTRAL, the Foundation may propose a modification (“proposed Modification”) to this Grant Agreement to change the Grant Term and the Report Deadlines, which proposed Modification shall be deemed approved unless the Grantee objects within 30 days of the transmission of the proposed Modification to Grantee. Any such proposed Modification shall be transmitted by the Foundation to Grantee via the Correspondence link on proposalCENTRAL and any such objection shall be transmitted by Grantee to the Foundation via the Correspondence link on proposalCENTRAL.

12. <u>Future Funding</u> By funding this grant, the Foundation assumes no obligation to provide other or additional support for the Grantee for any purpose. This Grant Agreement is not to be construed as an obligation to provide annual or other periodic donations to the Grantee.

13. <u>Liability</u> None of the Foundation, its trustees, directors, employees or agents shall incur any liability or bear any responsibility relating to or arising out of the Grantee's use, application, or expenditure of the grant funds.

14. <u>Modification</u> This Grant Agreement may be modified only by written agreement of the Foundation and the Grantee.

15. <u>Entire Agreement</u> This Grant Agreement constitutes the entire agreement among the parties and supersedes all prior written or oral statements or agreements.

16. <u>Notices</u> Unless specifically provided to the contrary, any notice required under this Grant Agreement will be in writing and sent by (i) registered or certified mail, (ii) a nationally recognized courier or delivery service or (iii) fax transmission, which notice shall be addressed to the parties at the addresses set forth at the end of this Grant Agreement. Such addresses may be changed by notice to the other party given in the same manner as provided above. Any notice shall be deemed received upon the actual delivery thereof.

17. <u>Signatures</u> A copy of this Grant Agreement, or any signature page hereto, transmitted via facsimile, proposalCENTRAL, or other means electronic transmission shall have the same force and effect as though it were an original.

[Signature page to follow]

FOUNDATION:

Craig H. Neilsen Foundation
16830 Ventura Boulevard, Suite 352
Encino, CA 91436
Facsimile: (818) 924-4286

By: ______________________________ ______________
Gordon R. Kanofsky Date
Co-Trustee, Craig H. Neilsen Foundation

GRANTEE:

Grant Recipient Organization: Centre Intégré Universitaire de Santé et de Services Sociaux de la Capitale-Nationale

______________________________ ______________
Signature of Authorized Signing Official Date

Print Name: ______________________ Title: ______________________

Street Address: ______________________________

____

Office Number: ______________________________

Facsimile: ______________________________

Email: ______________________________

Applicant: Best, Krista

Title: Evaluation of a Smartphone-based Peer Counselling program for individuals with spinal cord injury.

Program: PSR Postdoctoral Fellowships

Institution: CIUSSS-CN

App #: 366605

View Review Information

Close Window Print

Committee: PSR 2016 FGA

Summary Statement

Text Admin Summary:

CHNF has conditionally approved funding of this proposal. Please see the reviewer comments for a listing of the strengths and weaknesses identified in your application. The following issue(s) deserve particular attention: The proposed Smartphone-based Peer Counseling (SPC) program takes an interesting but simple, cost-effective approach to the complicated health problem of physical inactivity. While the technology is not entirely novel, reviewers noted that demonstrating the feasibility and potential effect of SPC through this project could justify a larger multisite study. While the study was ambitious, the applicant is well qualified, and the research design is logical and justified. The primary and secondary mentors identified in the training plan are both highly regarded in the field and will provide complementary expertise and training. Overall, the project will support the Fellow's growth as an independent and productive researcher in the field of PSR and SCI. Reviewers had two main concerns about the training plan: 1) To strengthen the Fellowship experience, it was strongly recommended that the training plan be updated to include additional coursework on the psychological aspects of peer counseling, motivation and self-efficacy; and 2) Additional detail about the different training roles of the two mentors needs to be added. As part of the due diligence process, the applicant is asked to address these two concerns and submit an updated training plan for programmatic approval.

Reviewer Role: Primary (237823)

OVERALL IMPACT

After considering all of the review criteria, summarize the significant strengths and weaknesses of the application and state the likelihood that Neilsen Foundation funding of this project will allow this Postdoctoral Fellow to become an important contributor to the field of SCI research.

:

.The project is interesting and innovative. The senior mentor is outstanding while the primary mentor can assist with training on issues of wheelchair mobility and research training. The applicant's qualifications are very good. The environment at CIRRIS is excellent for research training.. Concerns with the complexity of the methodology proposed are expressed.The training plan should allow the candidate to get some experience in learning about psychosocial issues related to peer counseling. A course or class on the psychological aspects of counseling, motivation and self-efficacy would provide the candidate with a broader understanding of these issues.

SIGNIFICANCE

1. Does the project address an important problem or a critical barrier in the field?

2. If the Aims of the project are achieved, how would this work change or enhance current methods, technologies, treatments, services, or interventions?

3. How will the Fellow's scientific knowledge, technical capability, and/or clinical skills be improved by undertaking this project?

:

The project address an important issue, that of physical activity (PA) in SCI and proposes to use an innovative approach based on smartphone based peer counseling (SPC). There are 3 main objectives involving development, measurement and feasibility but it is unclear how these may relate to a broader goal of developing and evaluating a tele health program to modify PA behavior. The development of the smartphone approach on itself if achieved can enhance current treatments and contribute to future interventions. However, the lack of methodological clarity and its relation to this broader goal casts doubts about the significance of this study.
It is assumed that the fellow will learn to develop an innovative intervention to improve PA. However, this proposal lacks specific information about how the fellow will benefit from conducting this project during her training.

RELEVANCE

The mission of the Craig H. Neilsen Foundation is "to improve the quality of life for those living with spinal cord injury and to support scientific exploration for effective therapies and treatments leading to a cure."

The overall goal for the new Psychosocial Research (PSR) initiative is to lead to "better outcomes for people living with SCI." Emphasis is placed on research directed towards:
a) increased understanding of psychological, social and environmental determinants of health, functioning and activity participation;
b) rehabilitation and habilitation interventions to improve psychological and social functioning, including participation in work, school and other community activities;

c) improved measurements of psychological, social and environmental risk factors, protective factors, processes and outcomes; and
d) identification of critical service gaps, needed data and/or new areas of exploration, within a psychosocial or socioecological context, as defined by, or with input from, people living with SCI.

1. How is this project relevant to the mission of the Foundation?

2. How is this project relevant to the goal of the Psychosocial Research initiative?

:
The proposed project is relevant to the mission of the Craig Nielsen Foundation by proposing an innovative intervention to improve physical activity after SCI. If the project objectives are achieved it will improve our understanding of the determinants of physical activity and will contribute to our current knowledge based by proposing a new intervention to peer counseling. The application lacks specific details about the relevance of this project as part of fellowship training.

INVESTIGATOR(S)
1. Are the PI, collaborators, and other contributors well suited to the project?

2. Does the FELLOW have the potential to develop as an independent and productive researcher?

3. Are the MENTOR/SPONSOR(S)' RESEARCH QUALIFICATIONS, including research support and track record of mentoring, appropriate for the proposed Fellowship?

4. Is there evidence of a match between the research interests of the Fellow and the mentor/sponsor(s) and is there a demonstrated ability and commitment to assist in assuring the Fellow's success?

5. Do the Research and Training Plans provide the Fellow with the requisite individualized and supervised experiences that will develop his/her research skills and serve as a foundation for a productive career?

:
1. The applicant is well suited to the project and to benefit from post-doctoral training. She has a Ph.D in rehabilitation sciences from UBC and a master's degree in Kinesiology. Areas where her knowledge could be improved by this training include those of greater psychosocial relevance including her outcomes of interest: motivation, self-efficacy, and satisfaction with psychological needs, and others as listed. However, these are not discussed in relation to training.
2. The applicant has 13 publications listed of which 6 she is the primary author. This suggests a strong potential to become an independent investigator and productive researcher.
3. The primary mentor, Dr. Routhier is an Assistant Professor and appears to be well qualified from a technical viewpoint. His background, however, is in mechanical engineering, rehabilitation and geriatrics, biomedical engineering and health administration and less so in psychosocial matters. Much of his work has been in wheelchair mobility. The second mentor, Dr. Luc Noreau, is well recognized nationally and internationally and can complement these areas of mentoring based on his background and reputation.
4. There seems to be a good match between the fellow and her proposed mentors' areas of interest and expertise. Both mentors provide supportive letters of recommendation showing their commitment to this fellow's education.
5. There is no detailed information about the mentors' roles in training for this fellowship.

INNOVATION

1. Does the project challenge and seek to shift current research or clinical practice/program intervention paradigms by utilizing novel theoretical concepts, approaches or methodologies, instrumentation, or interventions?

2. And/or does this application apply concepts, approaches or methodologies, instrumentation, or interventions from another field of research to spinal cord injury?

3. And/or is a refinement, improvement, or new application of theoretical concepts, approaches or methodologies, instrumentation, or interventions proposed?

:

1. The approach proposed is not completely novel but it does utilize new technology and a new approach to peer counseling.
2. Using smartphones to encourage behavioral changes has been adopted with other chronic disease populations such as in the fields of diabetes and cardiovascular disease.
3. The project proposes the adaptation of this technology for SCI.

APPROACH

1. Is the Research Plan of high scientific quality and does it relate to the applicant's Training Plan? Are the overall strategy, methodology, and analyses well-reasoned and appropriate to accomplish the Specific Aims of the project?

2. Are preliminary data provided to support the feasibility of the project and/or are potential problems, alternative strategies, and benchmarks for success presented? Or, if the project is in the early stages of development, will the strategy establish feasibility and will particularly risky aspects be managed adequately?

3. If the project involves clinical and/or community-based research: 1) are the plans for protection of human subjects from research risks described and adequate; and 2) are the plans for recruitment of patients/participants appropriate?

:

The overall approach is briefly described and conceptualized into four distinct phases of development, measurement, feasibility leading to a RCT which is not part of this project. The approach uses a mixed method design.
The first phase (development) proposes the use of focus groups with professionals and consumers, reviews of literature.
The second phase, measurement, uses a Delphi survey method to examine the proposed intervention and achieve 70% consensus. Next it will use a cross sectional design with within subjects comparisons to validate the primary outcome, actigraphy, in this study. 20 participants will be tested using their wheelchairs and examining the ability to propel themselves using both hands. Data analysis procedures are adequate for this phase although the effect of attrition or missing data is not accounted for.
The third phase, feasibility, will use a pre-posttest design to assess the feasibility of SPC investigation and other study outcomes. Study outcomes will be collected at 6 months post baseline and 3 month post intervention. The rationale for these time points is unclear plus contamination could occur between selected time points. Is 3 month follow up sufficient to demonstrate some of the selected outcomes such as increased motivation, satisfaction with participation and others. The design of this phase is not clear and lacks details in relation to the overall purpose of the study.
The discussion of participants' selection in relation to PA is also unclear. There is no mentioning of neurological classification or presence of comorbidities without much in-depth thinking about the relationship among these outcomes. Furthermore, the proposal seems overtly ambitious. For example, the applicant proposes also to explore

various delivery mediums for the SPC intervention. A large number of outcomes are proposed (i.e. anxiety, depression, and social support) but their relation to PA is not clearly described. A Figure depicting the conceptual model of the proposed approach would have been most beneficial in describing the approach. Last, the sample size for phase 3 is too small given the number of variables selected. Effect sizes are discussed without specific calculations based on outcomes. Overall, the proposed approach as described is unclear.
2. There is no preliminary data relevant to the project described.
3. While the plans for recruitment of participants are described, there is no clear mentioning of using informed consents and ways of protecting confidentiality. There is a small section on safety which does not describe specific strategies to be adopted.

ENVIRONMENT
1. Is the institutional environment for the scientific development of the Fellow of high quality, and is there appropriate institutional commitment to fostering the Fellow's training?

2. Will the research training environment provide the applicant with individualized and supervised experiences that will develop research skills needed in preparation for his/her research career?

3. Are the institutional support, physical equipment and other resources available to the investigators adequate for the project proposed?

4. Will the project benefit from unique features of the environment, subject populations, or collaborative arrangements?

:
It appears that the fellow will be placed at CIRRIS, a research center in Quebec City. Based on this information the environment will be conducive to her study and training. The institution seems committed and both mentors submitted letters of support.

NON-SCORED CRITERIA
(PLEASE NOTE THAT THE SCORE FOR THIS NON-SCORED SECTION MUST BE A "50 - NON-SCORED." PLEASE DISREGARD SCORING OPTIONS 1-9 FOR THIS SECTION ONLY.)

Please provide any important or relevant comments on each of the 4 non-scored criterion below.

1. Budget

2. Ethics/Safety

3. Other

4. Additional Comments for the Applicant

5. RESUBMISSION: When reviewing a Resubmission, the committee will evaluate the application as now presented, taking into consideration the responses to comments from the previous scientific review group and changes made to

the project.

:

1. The budget seems appropriate and there are no concerns.
2. IRB approval is not discussed.
3. The training plan is missing. This is a concern since this is a resubmission and this issue was pointed out during the first submission.

Reviewer Role: Secondary (237821)

OVERALL IMPACT

After considering all of the review criteria, summarize the significant strengths and weaknesses of the application and state the likelihood that Neilsen Foundation funding of this project will allow this Postdoctoral Fellow to become an important contributor to the field of SCI research.

:

This is an ambitious application that is justified adequately and proposed by a highly qualified applicant. The smartphone based peer counseling program is moderately novel by proposing to use a peer-coach and smartphone technology to deliver a physical activity intervention. The applicant proposes a scoping review, cross sectional study to validate the primary outcome of interest for measuring physical activity, and evaluate the feasibility of the intervention in the target population. Excellent mentoring resources are available to the applicant.

SIGNIFICANCE

1. Does the project address an important problem or a critical barrier in the field?

2. If the Aims of the project are achieved, how would this work change or enhance current methods, technologies, treatments, services, or interventions?

3. How will the Fellow's scientific knowledge, technical capability, and/or clinical skills be improved by undertaking this project?

:

This project would provide modest enhancement of current methods in physical activity promotion for people with SCI. Completing the project would provide the applicant with enhanced technical capability and skills in conducting technology-enhanced behavioral change research.

RELEVANCE

The mission of the Craig H. Neilsen Foundation is "to improve the quality of life for those living with spinal cord injury and to support scientific exploration for effective therapies and treatments leading to a cure."

The overall goal for the new Psychosocial Research (PSR) initiative is to lead to "better outcomes for people living with SCI." Emphasis is placed on research directed towards:
a) increased understanding of psychological, social and environmental determinants of health, functioning and activity participation;

b) rehabilitation and habilitation interventions to improve psychological and social functioning, including participation in work, school and other community activities;
c) improved measurements of psychological, social and environmental risk factors, protective factors, processes and outcomes; and
d) identification of critical service gaps, needed data and/or new areas of exploration, within a psychosocial or socioecological context, as defined by, or with input from, people living with SCI.

1. How is this project relevant to the mission of the Foundation?

2. How is this project relevant to the goal of the Psychosocial Research initiative?

:
The project is relevant to the mission of the Foundation by seeking to enhance outcomes for people living with SCI. It is relevant to the goal of the Psychosocial Research initiative by seeking to develop rehabilitation interventions to improve the psychological and social functioning of persons with SCI by enhancing their physical and social activity.

INVESTIGATOR(S)
1. Are the PI, collaborators, and other contributors well suited to the project?

2. Does the FELLOW have the potential to develop as an independent and productive researcher?

3. Are the MENTOR/SPONSOR(S)' RESEARCH QUALIFICATIONS, including research support and track record of mentoring, appropriate for the proposed Fellowship?

4. Is there evidence of a match between the research interests of the Fellow and the mentor/sponsor(s) and is there a demonstrated ability and commitment to assist in assuring the Fellow's success?

5. Do the Research and Training Plans provide the Fellow with the requisite individualized and supervised experiences that will develop his/her research skills and serve as a foundation for a productive career?

:
The applicant and collaborators are experienced and well suited to the project; the fellow application appears to have the potential to develop as an independent and productive researcher given her training and experience. Mentors have prior experience with early career trainees. There is a good match between the interests of the fellow and mentors.

INNOVATION
1. Does the project challenge and seek to shift current research or clinical practice/program intervention paradigms by utilizing novel theoretical concepts, approaches or methodologies, instrumentation, or interventions?

2. And/or does this application apply concepts, approaches or methodologies, instrumentation, or interventions from another field of research to spinal cord injury?

3. And/or is a refinement, improvement, or new application of theoretical concepts, approaches or methodologies, instrumentation, or interventions proposed?

:

There is a large and growing body of work on physical activity promotion interventions for people with physical disabilities, including SCI. The application is moderately novel by seeking to integrate technology with peer counseling. Completion of this project will provide the applicant with experience and preliminary data to pursue larger funding sources.

APPROACH

1. Is the Research Plan of high scientific quality and does it relate to the applicant's Training Plan? Are the overall strategy, methodology, and analyses well-reasoned and appropriate to accomplish the Specific Aims of the project?

2. Are preliminary data provided to support the feasibility of the project and/or are potential problems, alternative strategies, and benchmarks for success presented? Or, if the project is in the early stages of development, will the strategy establish feasibility and will particularly risky aspects be managed adequately?

3. If the project involves clinical and/or community-based research: 1) are the plans for protection of human subjects from research risks described and adequate; and 2) are the plans for recruitment of patients/participants appropriate?

:

The research plan is detailed and organized logically. The scheduled of tasks appears to be reasonable. The rationale for the fellowship are justified adequately. The applicant provides preliminary data to justify the proposed work. Plans to recruit human subjects and protect them adequately are described.

ENVIRONMENT

1. Is the institutional environment for the scientific development of the Fellow of high quality, and is there appropriate institutional commitment to fostering the Fellow's training?

2. Will the research training environment provide the applicant with individualized and supervised experiences that will develop research skills needed in preparation for his/her research career?

3. Are the institutional support, physical equipment and other resources available to the investigators adequate for the project proposed?

4. Will the project benefit from unique features of the environment, subject populations, or collaborative arrangements?

:

The clinical research provides the subject population and other resources needed to conduct this project successfully.

NON-SCORED CRITERIA

(PLEASE NOTE THAT THE SCORE FOR THIS NON-SCORED SECTION MUST BE A "50 - NON-SCORED." PLEASE DISREGARD SCORING OPTIONS 1-9 FOR THIS SECTION ONLY.)

Please provide any important or relevant comments on each of the 4 non-scored criterion below.

1. Budget

2. Ethics/Safety

3. Other

4. Additional Comments for the Applicant

5. RESUBMISSION: When reviewing a Resubmission, the committee will evaluate the application as now presented, taking into consideration the responses to comments from the previous scientific review group and changes made to the project.

:
No response entered

Détails du dossier - Décision

Numéro du dossier : 32092

Nom du candidat Best, Krista
Dossier 32092
Titre Active Living Lifestyles for Manual Wheelchair Users: A peer-delivered telephone approach.
Programme Formation postdoctorale pour les candidats non-résidents du Québec

Directeurs/codirecteurs de recherche ou Directeur du département

Courriel	Prenom, Nom
Luc.Noreau@rea.ulaval.ca	Noreau, Luc
Francois.Routhier@rea.ulaval.ca	Routhier, François

Acceptez-vous la bourse qui vous est offerte pour cette demande ? Oui

Etes-vous récipiendaire d'une bourse d'un autre organisme avec comités d'experts pour la durée de l'octroi offert ? Non

NOTE: Si oui, faire parvenir au FRQS une copie de l'offre de l'autre organisme.

Si vous ne désirez pas refuser l'octroi pour toute la durée offerte (ex. 1 année seulement), veuillez accepter l'octroi et inscrire les détails supplémentaires, relatifs à votre décision à la page "Informations supplémentaires".

Si vous êtes récipiendaire en 2015-2016 d'une bourse d'un autre organisme avec comité d'experts, le FRQS vous octroiera la différence dans le cas d'une bourse moindre que celle-ci. Vous devez donc accepter l'octroi offert.

NOTE: Vous aurez accès à la suite du dossier uniquement si vous acceptez l'octroi offert.

L'octroi est accepté par le candidat mais demeure conditionnel à la réception de certains documents :
Vous devez nous faire parvenir un document officiel attestant l'obtention de votre doctorat (par courriel en format PDF).
Votre directeur de formation postdoctorale doit nous confirmer par courriel le début de vos travaux de recherche (lyse.bourbonnais@frq.gouv.qc.ca).

Autorisations émises

Les montants ci-dessous sont fournis à titre informatif uniquement et peuvent différer si des modifications ont été apportées à votre dossier par le FRQS.

Année	Montant offert	Montant modifié	Conditionnel
2015/2016	30000		Oui
2016/2017	30000		Oui

Informations supplémentaires

Informations complémentaires pour la gestion de fichiers (le cas échéant)

Informations - Bourses de formation

Numéro d'assurance sociale : 125706051

DÉBUT DE LA BOURSE :

Si votre formation se poursuit au Canada, un chèque vous sera posté le 15 du mois; ce chèque couvre le mois complet. Les versements sont acheminés directement à l'adresse du laboratoire d'accueil du candidat indiqué à la page Informations financières. Veuillez indiquez le mois auquel vous aimeriez que débute votre bourse (entre mai et octobre 2015). Si votre bourse est conditionnelle, le paiement débutera lorsque la condition sera remplie. Un candidat ne peut reporter la date d'entrée en vigueur de sa bourse au-delà du 31 octobre 2015.

Mois du début de la bourse : Juillet

À quel endroit désirez-vous recevoir vos relevés d'impôt :

À l'adresse mentionnée ci-dessous.

**Adresse**	178 Kaye Street
**Ville**	Lr Sackville
**Pays**	CANADA
**Province**	Nouvelle-Écosse
**Code postal**	B4C 1N2

Mode de paiement

Si votre formation se poursuit à l'extérieur du Canada, vous aurez un chèque à tous les trois mois, c'est-à-dire quatre versements durant l'année. Nous pouvons faire parvenir vos chèques à votre laboratoire d'accueil sous forme de traites bancaires ou les poster à votre insitution financière au Québec.

Si votre formation se poursuit au Canada, choisir "Ne s'applique pas" parmi les choix offerts

Ne s'applique pas

Engagement en matière d'éthique

Identification des projets ou des programmes impliquant des sujets humains

Depuis mars 2008, Le Fonds de recherche du Québec - Santé (FRQS) exige que les détenteurs de bourses et de subventions acquièrent une formation de base en éthique de la recherche dans la mesure où leur projet ou leur programme de recherche porte sur des sujets humains.

Le terme « projet de recherche portant sur des sujets humains » inclut les projets réalisés :

Sur des sujets humains vivants;
Sur des cadavres, des embryons, des fœtus, des restes humains ou des tissus, y compris les liquides, gamètes, cellules ou du matériel génétique;
À partir de renseignements personnels contenus dans des dossiers.

Dans ce contexte et considérant cette définition, veuillez répondre à la déclaration suivante :

(En cas d'incertitude, veuillez valider cette information avec le comité d'éthique de la recherche de votre institution.)

Je déclare que mon projet ou programme de recherche, subventionné par le FRQS, porte sur des sujets humains.

Oui

Si vous avez répondu Oui à la question précédente:
Pour une bourse de formation :
Vous devez compléter la formation - niveaux 1 et 3 en cliquant sur le lien "programme de formation en éthique de la recherche". Suite à cette formation, vous devrez retourner par courriel, le certificat en format PDF au soin de la responsable du programme. La réception de ce certificat de formation est obligatoire pour que le FRQS autorise le paiement de votre deuxième année de financement.
Pour une bourse de carrière :
Vous devez compléter la formation - niveaux 1 et 3 en cliquant sur le lien "programme de formation en éthique de la recherche". Suite à cette formation, vous devrez retourner par courriel, le certificat en format PDF au soin de la responsable du programme. La réception de ce certificat de formation est obligatoire pour que le FRQS autorise le paiement de votre deuxième année de financement.
De plus, les chercheurs de carrière doivent participer comme observateur, à une réunion d'un comité d'éthique de la recherche (CER) rattaché à une université ou à un établissement du réseau de la santé et des services sociaux. Cette participation doit être réalisée dans les deux ans suivant la réponse positive du FRQS concernant leur bourse. Une lettre attestant de votre participation comme observateur, doit être émise par le CER et être expédiée au FRQS en format PDF. Cette participation doit être renouvelée lors de chaque octroi de bourse de carrière.
Pour une subvention de recherche :
Vous devez compléter (ainsi que tous les cochercheurs - excluant les collaborateurs) la formation - niveaux 1 et 3 en cliquant sur le lien "programme de formation en éthique de la recherche ". Au fur et à mesure que cette formation sera complétée, vous, ainsi que tous les cochercheurs, devrez retourner séparément par courriel, avant le début des versements, les certificats en format PDF, au soin de la responsable du programme. La réception de ces certificats de formation est obligatoire pour que le FRQS autorise le paiement de votre première année de financement.
Engagement du signataire
Le soussigné déclare et atteste ce qui suit :

J'atteste que tous les renseignements fournis sont exacts et que j'ai vérifié auprès des autorités compétentes, le cas échéant, avant de faire ma déclaration ci-dessus.

Si mon projet ou programme de recherche porte sur des sujets humains, je m'engage à fournir un certificat attestant que je me suis conformé aux exigences requises en matière d'éthique, telles que stipulées dans les conditions d'admissibilité du programme (disponibles sur le site web du FRQS), de même que chacun des cochercheurs au dossier s'il s'agit d'une subvention de recherche.
J'autorise le FRQS à échanger tous les renseignements concernant mon dossier pour fins d'études ou d'évaluation, sous la condition que les personnes ayant accès à ces renseignements en respectent le caractère confidentiel.
Je conviens que le présent engagement soit régi et interprété en vertu des lois applicables du Québec.

J'accepte : Oui

Informations financières

Laboratoire d'accueil

**Établissement**	Centre interdisciplinaire de recherche en réadaptation et intégration sociale de l'IRDPQ (CIRRIS)
**Adresse**	525 blvd Hamel
**Département**	Rehabilitation
**Au soin de**	Krista, Best
**Ville**	Quebec City
**Province**	Québec
**Pays**	CANADA
**Code postal**	G1M 2S8
**Affiliation universitaire**	Université Laval

Autorisations émises

Nombre	Année	Montant	Montant modifié	Statut	Conditionnel
1	2015/2016	30000		En cours	Oui
2	2016/2017	30000		En cours	Oui

Signature et transmission

Protection des renseignements personnels - CONSENTEMENT
Tous les renseignements de nature personnelle ou scientifique recueillis par le FRQS sont assujettis à la Loi sur l'accès aux documents des organismes publics et sur la protection des renseignements personnels (Loi sur l'accès). Le FRQS doit assurer le caractère confidentiel de ces renseignements et ne les communiquer qu'avec le consentement de la personne concernée ou conformément aux dispositions de la Loi.
Les données recueillies par le FRQS ont servi et serviront à : établir l'admissibilité des demandes; sélectionner les membres de comités d'évaluation; évaluer les demandes; assurer la gestion des programmes; procéder à des inventaires, des évaluations, des enquêtes, études ou analyses, dans le cadre de son mandat et conformément aux dispositions de la Loi sur l'accès à cet égard.
Ces activités peuvent nécessiter l'échange d'information avec les établissements de rattachement du demandeur. En acceptant le présent octroi, vous autorisez le Fonds de recherche du Québec - Santé à communiquer avec les établissements de rattachement concernés pour échanger tout renseignement relatif à la réalisation des activités énumérées précédemment.

Je m'engage à respecter les conditions émises à mon dossier pour bénéficier de l'offre d'octroi ;
Je m'engage à respecter les règles générales communes des Fonds de recherche du Québec et l'ensemble des conditions et exigences décrites sur le site web du FRQS ; Je m'engage à informer les autorités universitaires concernées, et directeur(s) de recherche s'il s'agit d'une bourse de formation. de l'offre d'octroi du FRQS ;
Je reconnais que seul le montant de l'année en cours constitue un engagement ferme de la part du FRQS. Les montants indiqués pour les années budgétaires subséquentes sont mentionnés à titre indicatif seulement. Ceux-ci peuvent être modifiés en tout temps par le conseil d'administration du FRQS, sans autre préavis, en fonction notamment des crédits qui lui sont alloués annuellement par l'Assemblée nationale du Québec, de ses priorités stratégiques et de son processus budgétaire.
J'accepte que le FRQS diffuse des données relatives à cet octroi, dont le nom du boursier ou du chercheur financé, le montant, le programme et le titre du projet, de même que le résumé vulgarisé soumis dans la demande, notamment par l'entremise de ses plateformes de communication (Web, réseaux sociaux, etc.), afin de promouvoir la recherche soutenue par des fonds publics québécois.
Je m'engage à adopter une conduite responsable en recherche et à respecter les dispositions de la Politique sur la conduite responsable en recherche du Fonds de recherche en Québec - Santé.. Advenant une allégation de manquement à la conduite responsable en recherche visant mes "activités de recherche", j'accepte que le rapport d'examen de la plainte (incluant les renseignements personnels me concernant), mené conformément aux exigences de la Politique des Fonds, soit communiqué par l'établissement aux FRQ (FRQNT, FRQS, FRQSC) afin que les Fonds puissent prendre des mesures appropriées en cas de manquement avéré. Ces mesures peuvent inclure des sanctions relatives au financement des FRQ ou à mon admissibilité à recevoir un tel financement. Si une intervention urgente était nécessaire pour prévenir ou cesser un dommage avant la fin du processus d'examen, l'établissement pourra également communiquer les informations relatives à l'allégation aux Fonds.
Je m'engage à aviser immédiatement le Fonds si je deviens non admissible à faire une demande de financement ou à détenir des fonds de recherche publics d'une agence publique de financement de la recherche au Canada ou à l'étranger, en raison d'un manquement avéré à la conduite responsable en recherche. La poursuite du soutien financer des Fonds pourra alors faire l'objet d'un examen par le comité en conduite responsable en recherche des FRQ.

Je m'engage à faire valoir la contribution financière du FRQS dans toute publication et document officiels réalisés grâce à cet octroi.

Krista Best

J'accepte: Oui
